# BP180 Is Critical in the Autoimmunity of Bullous Pemphigoid

**DOI:** 10.3389/fimmu.2017.01752

**Published:** 2017-12-08

**Authors:** Yale Liu, Liang Li, Yumin Xia

**Affiliations:** ^1^Department of Dermatology, The Second Affiliated Hospital, School of Medicine, Xi’an Jiaotong University, Xi’an, China; ^2^National-Local Joint Engineering Research Center of Biodiagnostics and Biotherapy, The Second Affiliated Hospital, School of Medicine, Xi’an Jiaotong University, Xi’an, China

**Keywords:** BP180, bullous pemphigoid, autoantibody, dermal–epidermal junction, cytokine

## Abstract

Bullous pemphigoid (BP) is by far the most common autoimmune blistering dermatosis that mainly occurs in the elderly. The BP180 is a transmembrane glycoprotein, which is highly immunodominant in BP. The structure and location of BP180 indicate that it is a significant autoantigen and plays a key role in blister formation. Autoantibodies from BP patients react with BP180, which leads to its degradation and this has been regarded as the central event in BP pathogenesis. The consequent blister formation involves the activation of complement-dependent or -independent signals, as well as inflammatory pathways induced by BP180/anti-BP180 autoantibody interaction. As a multi-epitope molecule, BP180 can cause dermal–epidermal separation *via* combining each epitope with specific immunoglobulin, which also facilitates blister formation. In addition, some inflammatory factors can directly deplete BP180, thereby leading to fragility of the dermal–epidermal junction and blister formation. This review summarizes recent investigations on the role of BP180 in BP pathogenesis to determine the potential targets for the treatment of patients with BP.

## Introduction

Bullous pemphigoid (BP), by far the most common autoimmune blistering disease, is induced by autoantibodies against the structural components of the dermal–epidermal junction (DEJ) (1). In most cases, the disease develops cryptically (2). The suggested causes of BP include silicosis (3), psoralen and ultraviolet A therapy (4), infections (5), physical or chemical insults (6–8), certain fruits (9), and medications (10, 11). However, the validation of these factors in the pathogenesis of BP remains be established. BP mainly affects the older age group of both sexes, or those 70 years old and above, but it can also affect infants, children, and adolescents (1, 12). This disease mainly involves the skin but occasionally the eyes, mouth, and genitals (1, 2). The cutaneous manifestations of BP are polymorphic and can be classified into three groups, namely classical BP, non-bullous cutaneous pemphigoid, and various rare variants (13, 14). Classical BP is clinically characterized by large (1–3 cm), tense, serous, or hemorrhagic blisters that appear on erythematous, urticarial, or eczematous lesions and even on apparently normal skin (1, 13). The biopsied lesions exhibit subepidermal splitting or blisters, which is the hallmark of BP, with dense inflammatory infiltration of eosinophils, basophils, neutrophils, lymphocytes, and mast cells in the dermis (1). Immunofluorescence analysis is necessary for the diagnosis of BP (15). Direct immunofluorescence is the most sensitive method for BP diagnosis, in which the lesion shows linear deposition of immunoglobulin G (IgG), C3 complement, and even IgE at the DEJ (16–18). Indirect immunofluorescence using the patient’s sera and a substrate, especially salt-split skin, reveals a linear deposition of IgG along the roof of the artificial split (18).

One typical serologic characteristic of BP is the presence of circulating autoantibodies, which are mostly against BP180 (collagen XVII) and BP230 (15, 19, 20). BP180 is a 180 kDa transmembrane glycoprotein with a 16th non-collagenous (NC16A) domain, which is the immunodominant part in BP (14). BP230 is an intracellular constituent of the hemidesmosomal plaque and belongs to the spectraplakin family (20, 21). The autoantibodies reported in BP include IgG and IgE (1, 22). Usually, IgG autoantibodies to BP180 are the ones first to be detected, and then IgG autoantibodies to BP230 subsequently appear (23). IgE antibodies to BP230 can also be detected in the blood of BP patients (24). Given the existence of autoantibodies, there have been commercially available enzyme-linked immunosorbent assay (ELISA) kits that target BP180 and BP230 antibodies for BP auxiliary diagnosis (25, 26).

Due to the age group involved and the application of more sensitive and specific diagnostic assay systems, the reported BP morbidity has increased (14, 19, 27, 28). Moreover, for disease-specific factors, due to the concomitant occurrence of neurodegenerative disorders, use of higher doses of oral corticosteroids, and the propensity to malignancies and venous thromboembolism, BP mortality showed an increasing trend as well (19, 29–36). These findings suggested the contributory role of activation of blood coagulation in the pathogenesis of BP (35, 36). Presently, topical or systematic corticosteroids, with or without immunosuppressive agents, are still the mainstays for BP treatment (1, 14, 37, 38). Intravenous Ig has also been introduced as an alternative therapy for BP (39–41), however, its effectiveness is still questionable (42, 43). Therefore, it is of highly importance to discover new targets to reduce BP morbidity and mortality. Recently, increasing evidences show that autoimmune responses to BP180 are important in the initiation and evolution of BP (44). The binding of autoantibodies to BP180 is a central step for blister formation. Moreover, BP180 is associated with severe and extensive lesions that require higher dose of steroids, which is a key risk factor for death (14, 28, 45). The serum level of anti-BP180 NC16A autoantibody correlates with the more active and severe disease, as well as poorer prognosis (33, 46). We, thus, consider BP180 as the most important culprit in the pathogenesis of BP and focused this review on recently updated knowledge on BP180 and its autoantibodies in BP.

## The Basic Structure of BP180

BP180 is a type II transmembrane protein with a cytosolic NH2 terminal and an extracellular COOH domain (47). The N-terminal domain, transmembranous stretch, and extracellular C-terminus have 466, 23, and 1,008 amino acids (aa) in length, respectively (48). The ectodomain contains 15 collagenous subdomains (COL1–COL15) interspersed by 16 non-collagenous sequences (NC1–NC16). The NC16A domain, a juxtamembranous linker region, appears to be biologically important, as it serves as the nucleus for the formation of a collagen-like triple helix (49, 50). The extracellular domain contains coiled-coil structures, which are physiologically shed from the cell surface by a disintegrin metalloproteinase (ADAM) (50). The ectodomain forms a loop structure as it spans the lamina lucida, extends to lamina densa, and then kinks back into the lamina lucida (49). BP180 contains multiple binding sites for hemidesmosome proteins, including the extracellular domains of integrin α6 and laminin-332 (laminin-5) and the cytoplasmic domains of integrin β4, plectin, and BP230 (20). The structure and location of BP180 indicate that it acts as a core anchor protein that connects the intracellular and extracellular hemidesmosomal proteins and plays a key role in the pathogenesis of BP.

## The Epitope Profiles of BP180

**Figure 1:**
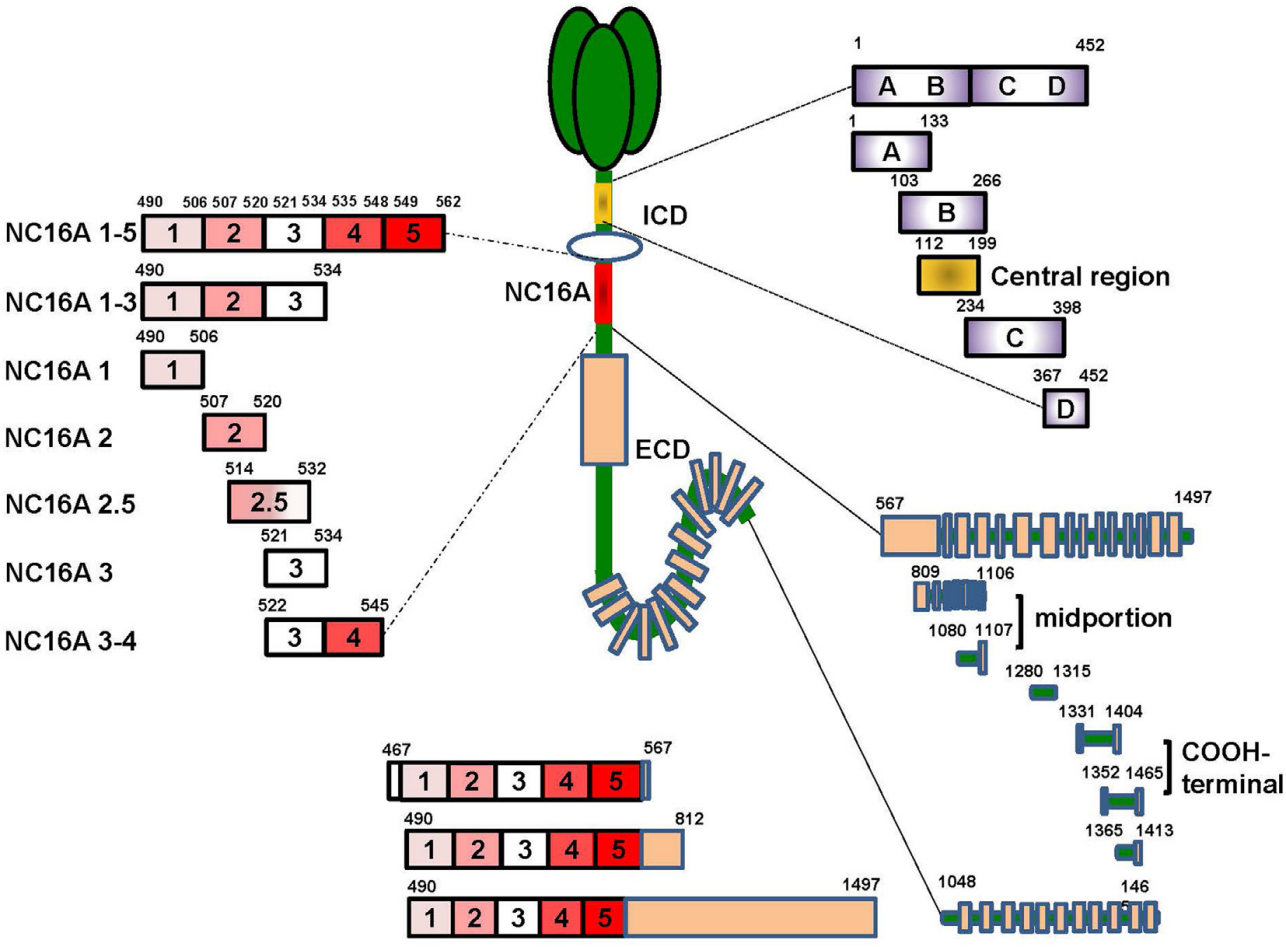
The target sites of the BP180 molecule. BP180 is a multi-epitope protein with three major domains-the intracellular domain (ICD), the NC16A domain and the ectodomain outside NC16A domain. The ICD include five target sites, including aa 1–452, aa 1–133, aa 103–266, aa 234–398, aa 36–452 and a central region aa 112–199. The NC6A domain contain seven targeted sites, that is, NC16A1-5, 1-3, 1, 2, 2.5, 3, 3-4. The ectodomain domain also have eight functional sites, namely aa 567–1497, aa 809–1106, aa 1080–1107, aa 1280–1315, aa 1331–1404, aa 1352–1465, aa 1365–1413, and aa 1048–1465. Additionally, there are also target sites crossing more than one domain, such as aa 46–567, aa 490–812, and aa 490–1497.

Previous studies mainly focused on extracellular NC16A domain (aa residues 490–562), which is the main target of BP autoantibodies. The NC16A domain has seven antigenic sites, including NC16A1, NC16A1-3, NC16A1-5, NC16A2, NC16A2.5, NC16A3, and NC16A3-4 (51–53) (Figure 1). Among these sites, NC16A2 and NC16A2.5 are the major antigenic sites, which can be targeted by all IgG and IgE antibodies. However, recent studies have described additional autoantibody-binding domains of BP180, such as the intracellular domain (ICD) and ectodomain (44, 54). The ICD (aa 1–452) has five target sites, namely ICD A, ICD B, ICD C, ICD D, and ICD A-D, and a central region (aa 112–199) (Figure 1). A previously published study reported that out of 18 sera of BP patients, 16 reacted with recombinant ICDs and that most of the antibodies bind to the central portion (55). A great number of sera combined with at least one of the ICD regions. With regard to ectodomain, it has been reported that 7.8–47% of BP sera recognized the C-terminal regions of the ectodomain (54, 56). Further mapping identified the six regions outside of NC16A that were recognized by the sera of the patients: aa 809–1106, aa 1080–1107, aa 1280–1315, aa 1331–1404, aa 1365–1413, and aa 1048–1465 (11, 52, 54, 57). aa 809–1106 and aa 1080–1107 were at the midportion, whereas aa 1331–1404 and aa 1365–1413 were at the COOH-terminal (Figure 1). Other epitopes embracing more than one domain, such as aa 467–567, aa 490–812, and aa 490–1497, were also reported (11, 52). It has been suggested that the pattern of epitope recognition may influence the course of the disease (23). Therefore, the recognition of target regions within BP180 is substantial in understanding the disease initiation and clinical characteristics of BP.

## The Source of Autoantibodies to BP180

The etiology of BP is complex, but the presence of autoantibodies was widely accepted as the *sine qua non* of the condition. Anti-BP180 autoantibodies also exist in healthy people, even though these antibodies are conformationally different from pathogenic ones; however, only those bound to skin basement membrane can induce BP—suggesting that autoantibodies in the healthy may not be pathological *per se* (58, 59). The autoantibodies may assume function of surveillance and self-tolerance (60). In pathologic conditions, self-tolerance of the autoantibodies is dysfunctional, thus leading to the production of a higher-level of autoantibodies that bind to skin basement membrane and give rise to the occurrence of BP. The development of BP suggests that there is a threshold or checkpoint in terms of autoantibody generation (61). It remains unclear why immune tolerance to BP180 is dysfunctional in some individuals. Previous study suggests that CD4+ CD25+ Foxp3+ regulatory T (Treg) cells play an indispensable role in maintaining self-tolerance and in suppressing excessive production of autoantibodies deleterious to the host (62–65).

**Figure 2:**
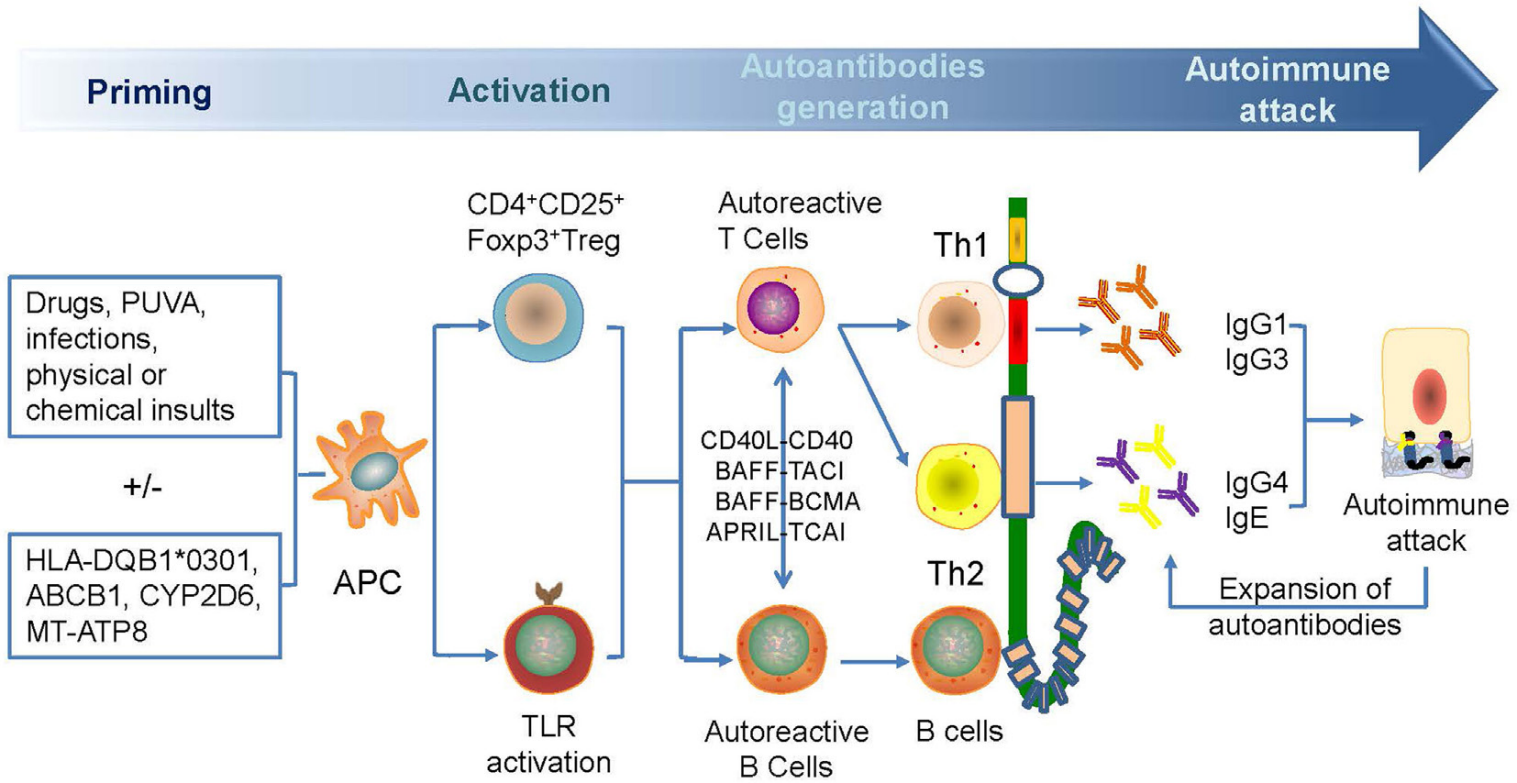
A possible mechanism for the generation of anti-BP180 autoantibodies. Antibodies are generated for the breakage of self-tolerance which is caused by drugs, psoralen, and ultraviolet A therapy, infections, physical, or chemical insults. Autoactivated Th1 and Th2 and B cell can target different domains of BP180, leading to the generation of anti-BP180 autoantibodies *via* epitope spreading and Ig class-switch. Such autoantibodies could be present in the serum for a long time before occurrence of clinical features. Attacked BP180 can be a source of new antigens to initiate the further expansion of autoantibodies and acceleration of disease.

The reduction of CD4+ CD25+ Foxp3+ Treg cells in BP, as induced by triggers that are variants of pre-existing genetic factors, such as HLA-BQB1*0301, CYP2D6, MT-ATP8, and so on, leads to the breakage of self-tolerance, followed by the increase in autoreactive Th2, Th1, and B cells that can recognize different domains of BP180 mediated by epitope spreading to produce different autoantibodies (14, 44, 59, 65–69). The pathogens can exacerbate the process by sensitizing B cells *via* binding to toll-like receptors. The autoreactive T cells can interact with autoreactive B cells *via* combinations of CD40L–CD40, B-cell activating factor–transmembrane activator and CAML interactor (TACI)/B-cell maturation antigen, and proliferation-inducing ligand–TACI to further break peripheral tolerance and induce Ig production and class switching (70–74) (Figure 2). Moreover, the reactivity of T and B cells that target the NH2-terminal portion of the BP180 ectodomain is associated with severe BP, whereas the crosstalk of T and B cells targeting the central portion of BP180 is more frequently recognized in limited BP (75). The exploration in gene therapy might provide clues to retrieve Treg-mediated tolerance and to hinder the production of autoantibodies in skin-grafted animals (76).

## Autoantibodies Targeting NC16A of BP180

Previously, most studies pointed out that the NC16A might be the major pathogenic epitope in BP (47, 74). ELISA analysis using recombinant BP180 NC16A demonstrated that 22–100% of BP sera reacted to BP180 NC16A peptides and that autoantibodies targeting NC16A domain are associated with tense blisters, severe urticarial erythema, extensive lesions, and elevated eosinophils (45, 77). Therefore, there is a variety of autoantibody types that act on this domain and mediate various pathogenesis.

### Anti-NC16A IgG

**Figure 3:**
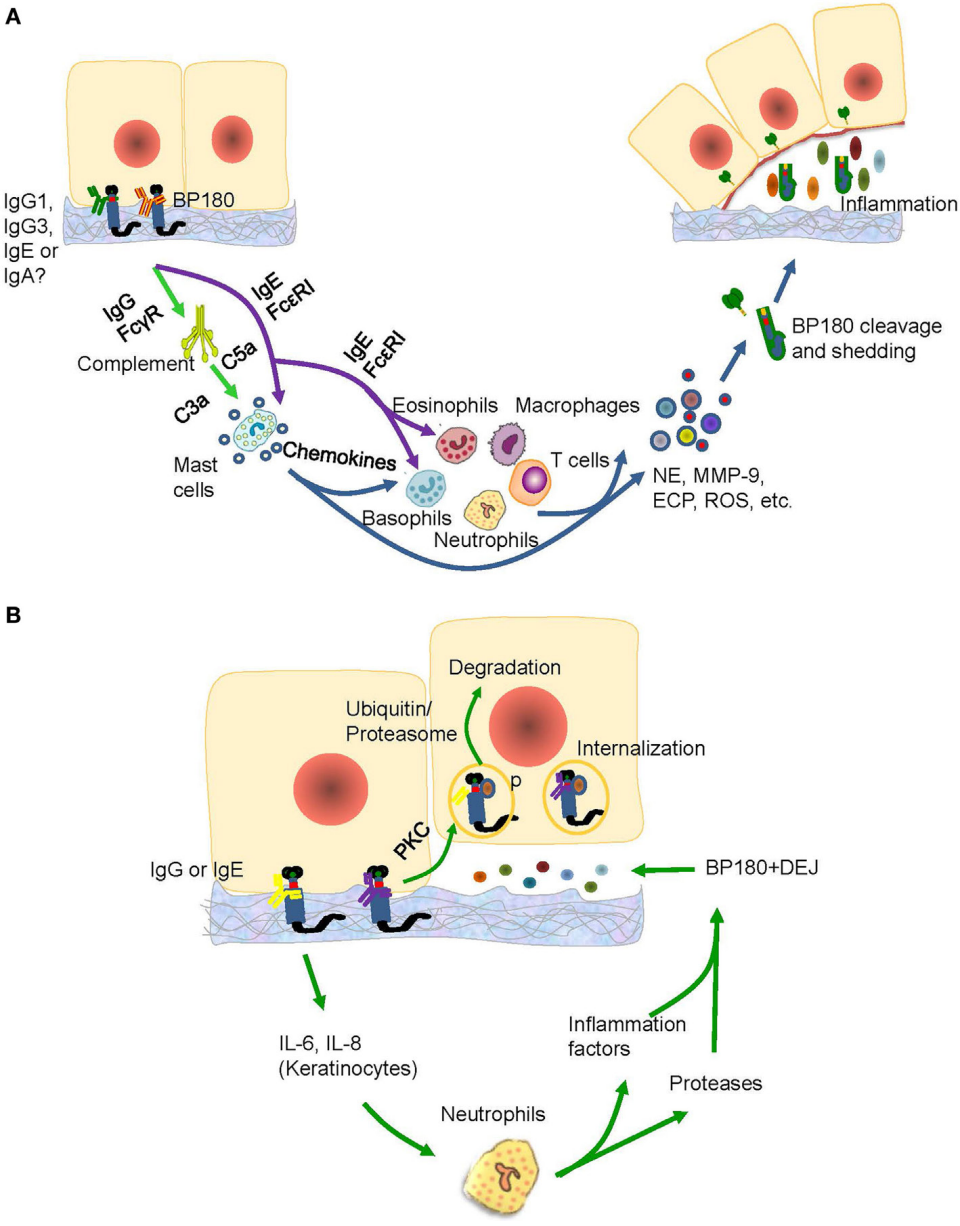
The possible pathogenic mechanism of the anti-BP180 NC16A autoantibodies. **(A)** The IgG1, IgG3, IgE, or IgA anti-BP180 NC16A autoantibodies-mediated pathways for blister formation in bullous pemphigoid (BP). The binding of IgG1, IgG3 with NC16A activates complements *via* FcγR followed by mast cell degradation and neutrophilic, eosinophilic, basophilic, and macrophage infiltration, which lead to the degradation of BP180 by releasing inflammatory factors and proteases. While the IgE anti-BP180 NC16A autoantibodies activate infiltration of mast cell, eosinophils, and basophils *via* FcεRI. **(B)** The immunoglobulin G (IgG) or IgE-mediated pathways for blister formation in BP. On the one hand, the binding of IgG or IgE with NC16A domain activates the protein kinase C followed by BP180 phosphorylation and degradation, which leads to the reduced adhesion. The binding, on the other hand, causes the release of interleukin (IL)-6 and IL-8 by keratinocytes, which prompts recruitment of neutrophils, release of inflammatory factors and neutrophil elastase (NE), and blister formation.

Anti-NC16A IgG is associated with BP-affected areas and with the occurrence of erosions and blisters in BP (46). High titers of anti-BP180 NC16A IgG at the time of therapy cessation represented the main factor in the prediction of risk of relapse in BP (78). Passive transfer of rabbit antimurine IgG antibodies against BP180 can lead to the development of BP-like skin phenotype, in which the mechanisms involved are complement activation, mast cell degradation, neutrophil infiltration, production of reactive oxygen species and proteases, and BP180 degradation (14, 79); and these mechanisms suggest a complement-dependent inflammatory pathway in BP development. The pathways induced by antimurine BP180 NC16A domain is further verified in studies using mast cell-deficient (80), C5-null (16), C4-null, alternative pathway component factor B-deficient (28, 81), membrane CD46 upregulated (82), Fab-IgG-deficient (83), and FcγR-deficient (84) mice. All these studies were able to identify the complement-dependent inflammatory pathway of anti-BP180 NC16A IgG (Figure 3A).

There are complement-independent mechanisms that account for the induction of BP by anti-NC16A IgG. Nearly one-fifth of BP cases may develop blisters in a complement-independent manner mainly through BP internalization (16) Immunofluorescence microscopy revealed that BP180 content in BP lesions is reduced by approximately 40% (85). As demonstrated by vibration assay *in vitro*, keratinocytes stimulated with anti-NC16A IgG demonstrated BP180 internalization and significant decrease in cell-plate adhesion (86). Further supporting data stem from an *in vivo* study using neonatal C3-deficient BP180-humanized mice without complement activation (87). The effects are attributed to the internalization of BP180/anti-BP180 complex via a macropinocytic pathway, which involves ICD phosphorylation by protein kinase C and potential degradation of BP180 through a ubiquitin/proteasome pathway (85, 88, 89). As BP-IgG-induced BP180 internalization is insufficient to induce blister formation, various inflammatory responses mediated by FcγR-independent and FcγR-dependent pathways must be involved, which further lead to a BP-specific split (85). At least interleukin (IL)-6 and IL-8, which are induced by autoantibodies, participate in the inflammatory responses (28, 90) In addition, neutrophils partly recruited by IL-8 are also essential for blister formation (91) (Figure 3B). These studies emphasized the complement-independent inflammatory pathway of anti-BP180 NC16A IgG.

However, the role of complements in BP pathogenesis, as mediated by anti-BP180 NC16A IgG autoantibodies, is still controversial. Negative C3 deposition along the epidermal basement membrane zone was found in 16.9% of BP lesions (16). Antihuman BP180 NC16A IgG4, which has low ability to bind to the Fc receptor and fixing complement, can induce dermal–epidermal separation in *in vitro* cryosection assays and blister formation in patients (89, 92). IgG4 autoantibodies are also the major IgG subclasses of autoantibodies found in more than 54.4% of BP patients, and it is parallel with the disease severity (93). An *in vitro* study found that anti-NC16A IgG4 might prevent the induction of BP blistering by competitively inhibiting the binding of IgG1 and IgG3 autoantibodies to the NC16A region and by blocking IgG1- and IgG3-induced complement fixation and neutrophil infiltration (94). Another study reported that anti-NC16A IgG4 has a protective role in BP (94). However, the provided C5a complement could successfully induce BP through anti-NC16A IgG4 (94). The revealed discrepancies may be explained by the different research methods used in the studies, as well as the complexity of BP, or by the possibility that the protective role of IgG4 autoantibodies in BP is due to the competitive blockade of IgG1 and IgG3 autoantibodies, which in turn gives rise to the suppression of complement-dependent blister formation. However, the “IgG4-dominant complement-independent BP” cannot be excluded. When the abovementioned studies are summarized, as well as the findings of complement fixation at basement membrane in BP patients, we can conclude that complement amplifies blister formation by inducing inflammation (16, 51, 95).

### Anti-NC16A IgE

In addition to the IgG autoantibodies, 22–100% BP patients also produce IgE autoantibodies against BP180 NC16A (24, 46, 96, 97). The level of anti-NC16A IgE is correlated with disease activity (24, 46), occurrence of urticarial lesions and erythema (46, 98, 99), higher prednisolone dosage, longer duration before remission, and more intensive therapies (100). Immunofluorescence revealed the deposition of IgE autoantibodies along the DEJ in up to 41% of BP patients (101). Moreover, the early pathological changes in BP, including urticaria, eosinophil infiltration, and spontaneous blistering, can only be observed in models that utilized IgE autoantibodies from patient sera or recombinant monoclonal IgE antibodies specific for BP180 (102). These observations indicate that IgE autoantibodies may also be involved in the pathogenesis of BP and correlate with certain distinct clinical features. Furthermore, epitope mapping studies have demonstrated that these IgE autoantibodies preferably target the NC16A domain of the BP180 protein as IgG (46, 53, 103).

Injecting purified anti-BP180 NC16A IgE autoantibodies into human skin grafted on nu/nu mice can induce histologic dermal–epidermal separation, as well as erythematous and urticarial plaques; and the mechanisms of these processes include mast cell infiltration and degranulation and influx of eosinophils, lymphocytes, and neutrophils (104). An *in vitro* investigation showed that the injection of IgE into the dermis of a human cryosection model led to histologic separation at the DEJ through the binding of FcεRI on mast cell surface, which triggered mast cell degranulation, subsequent eosinophil infiltration, and direct activation of eosinophils and basophils mediated by high-affinity FcεRI (95, 105, 106). Interestingly, the amount of circulating eosinophils is correlated with the levels of both NC16A-specific IgG and IgE in BP sera (106). These results provide indirect evidence that anti-BP180 NC16A IgE autoantibodies contribute to BP-like damage and to certain distinct clinical features by triggering mast cell degranulation and basophil histamine release that is FcεRI dependent (106, 107) (Figure 3A). The successful use of omalizumab in preventing the interaction of IgE with FcεRI in BP patients further verifies the FcεRI-dependent pathways (108, 109). However, recent studies also revealed that IgE autoantibodies from BP patients could be internalized into cultured human keratinocytes or skin tissues where they stimulate production of IL-6 and IL-8 and lead to the depletion of hemidesmosomes, as observed through BP IgG autoantibodies and as the effect of anti-NC16A IgG on keratinocytes *in vitro* (110–112) (Figure 3B). These studies suggest that the direct function of anti-BP180 NC16A IgE autoantibodies is to promote inflammation and fragility of the DEJ in BP. Further studies utilizing IgE monoclonal antibody are necessary to explore the mechanisms underlying NC16A-specific IgE autoantibody-mediated tissue damage in BP (113).

### Anti-NC16A IgA

An increasing number of studies reported the potential role of anti-BP180 IgA, aside from anti-NC16A IgG and IgE, in BP pathogenesis (52, 107, 114, 115). Comparable to IgG and IgE, IgA autoantibodies mainly target the NC16A domain (106). Anti-BP180 NC16A IgA can be found in sera of 20–65% of BP patients (51, 113); and it can also be detected in the saliva of 36%, parotid gland of 44%, and in sera of 28% of mucous membrane pemphigoid patients (114). Moreover, IgA basement membrane zone deposition has been reported in 13% of BP patients (17, 116). However, investigation that mechanistically elucidates the functions of IgA autoantibodies in BP are still lacking. Epitope spreading or antibody class switching are likely to be involved in the pathogenesis of BP, as there is a determined clinical association between BP and linear IgA bullous disease (LAD) (114, 117). Recent studies reported that there is a linear IgA deposition in basement membrane zone, which is dapsone-responsive and characterized by a flexural distribution of intensely pruritic subepidermal bullae, thus suggesting that IgA might be associated with specific clinical features of BP or that BP may have comparable or overlapping pathomechanisms with LAD (118, 119). Like LAD, the anti-BP180 IgA autoantibodies directly act on NC16A domain, leading to the release of inflammatory factors and neutrophils, degranulation of neutrophils and mast cells, and release of proteolytic enzymes—all of which are similar to the effects of IgG and IgE (118) (Figure 3A). In fact, most serum samples from LAD and BP patients contain both IgA and IgG antibodies against BP180 (114, 120, 121). Thus, the two diseases could be regarded as different ends of a continuous spectrum of autoimmune responses to BP180 in subepidermal blistering diseases (119). Further studies using cell and animal models are needed to comprehensively unveil the pathogenic role of anti-BP180 NC16A IgA autoantibodies.

## Autoantibodies Targeting ICD and Ectodomain of BP180

**Figure 4:**
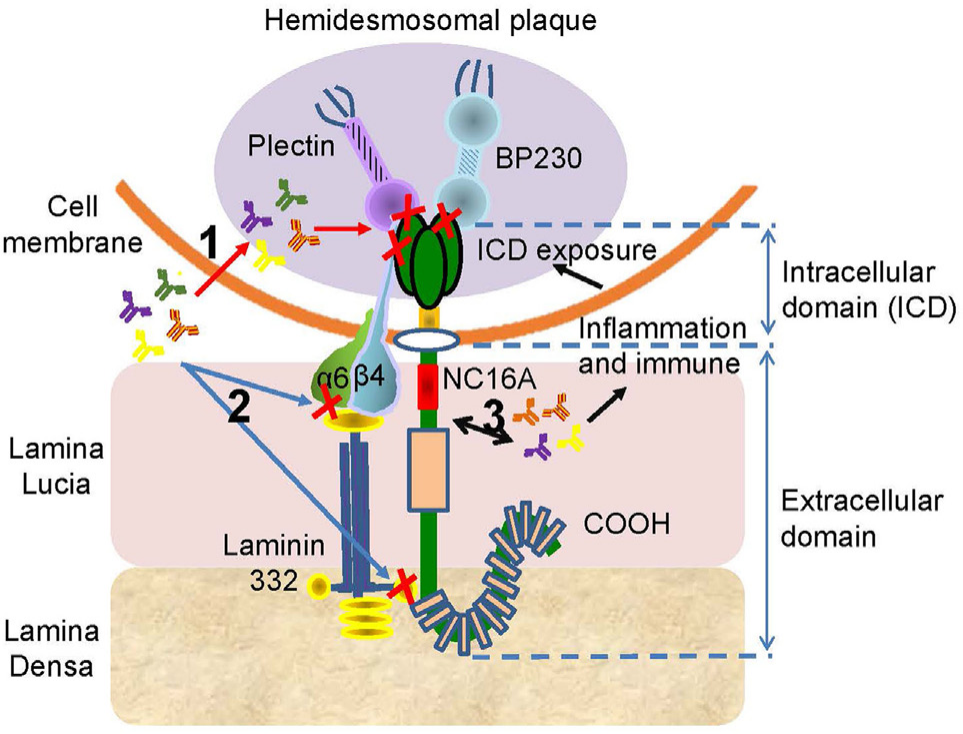
The potential pathogenesis of the anti-BP180 autoantibodies targeting the intracellular domain (ICD) or the ectodomain outside the NC16A. There are three possible mechanism associated the autoantibodies with ICD or ectodomain. (1) The autoantibodies penetrate cells, reach the ICD, and inhibit the interaction of BP180 with plectin, BP230, or β4. (2) The binding of autoantibodies with ectodomain interferes the interplay of BP180, α6, β4, and laminin-332. (3) The interaction between autoantibodies and ectodomain induces inflammatory and immune responses, which lead to the exposure of the ICD, initiating the effect of autoantibodies on ICD.

Recent studies reported that 59–82% of BP sera can recognize the ICD of BP180, while 7.8–49% of BP sera are reactive against the ectodomain of BP180 (54, 77, 122, 123). All autoantibodies, including IgG, IgE, and IgA, can target ICD; however, these autoantibodies bind to different sites (55, 114, 122, 123). The autoantibodies can penetrate live cells, reach their intracellular targets, and alter cellular functions (124) (Figure 4). The central region of BP180 ICD harbors binding sites that are critical for the interaction of BP180 with β4 subunit of the α6β4 integrin, which is vital for the incorporation of the protein into the hemidesmosome (49). Thus, it implicates that autoantibodies against BP180 ICD impair the interaction of BP180 with other molecular constituents of the hemidesmosome. Otherwise, the damaged basal keratinocyte induced by the binding of autoantibodies to BP180 ectodomain leads to the exposure of the ICD to the immune system, which is referred to as “epitope spreading” (125) (Figure 4).

In addition, the COOH-terminal region of the BP180 ectodomain is shown to be recognized by 47% of BP sera (56). IgG, IgE, and IgA autoantibodies can all bind to the terminal region (52, 54, 103, 122). The presence of autoantibodies against N- or C-terminal portions of the BP180 ectodomain is associated with the mucosal lesions in BP patients (56, 126). In addition, there are existing autoantibodies against the midportion of BP180; and these are associated with the occurrence of hemiplegia, clinical presentation of lack of erythema around the bullae, and histopathologic eosinophil infiltration inside and around subepidermal bullae (57). Other studies revealed that high levels of autoantibodies against C-terminal portions are associated with older age, administration of dipeptidyl peptidase-4 inhibitors before BP onset, and a positive response to moderate doses of oral prednisolone (11, 123). However, there is also a report refuting the association of autoantibodies with dipeptidyl peptidase-4 inhibitors (127). As BP180 extends from the cytoplasm of the basal keratinocyte to the lamina densa, it is presumed that the autoantibodies against this region might be responsible for the scarring phenotype observed in cicatricial pemphigoid patients (56) (Figure 4). The development of novel ELISA kits to detect the autoantibodies against the ectodomain, or even ICD, is beneficial in diagnosing BP without NC16A domain (56, 128).

More novel animal models have been recently constructed, thus making it possible to determine the role of different domains. One of the animal models is the ΔNC14A mice, which have BP180 NC14A replaced with the homologous human BP180 NC16A epitope cluster region (129). BP lesion develops in these ΔNC14A mice after passive transfer of BP IgG (129). The NC14A region can also be genetically deleted in C57BL/6 mice, which then have less amount of BP180 in skin but have normal ectodomain shedding (130). They spontaneously produce IgG and IgA autoantibodies against BP180 and present eosinophilic infiltrations, as well as the clinical features of pruritus and crusted erosions (130). Hence, the ΔNC14A mice may be an ideal experimental model for investigating the early clinical changes in BP. However, in the absence of NC16A domain, it is impossible to explore the detailed functions of anti-NC16A autoantibodies. It is also presumed that the pruritus and eosinophils are associated with the ectodomain. Therefore, the ΔNC14A mice may be utilized as a model for the exploration of autoantibodies acting on the ICD or on the ectodomain. However, the mechanisms involved remain to be confirmed. Another animal model is the COL17-humanized mice, which can express human BP180, and it is suitable for the analysis of the pathogenesis of BP in humans (131). The spontaneous production of high titers of anti-BP180 antibodies in blisters and erosions on erythematous skin lesions makes the observation of dynamic immune reactions possible. The pathogenicity of autoantibodies against ICD and ectodomain of BP180 remains unclear, and further studies are warranted. The development of novel ELISA system to detect such autoantibodies is necessary (77).

## IgM Autoantibodies in BP

An IgM-mediated BP has been recently reported (132, 133). Direct immunofluorescence microscopy showed that linear deposition of IgM can be found at the DEJ of 6–22% of BP patients (17, 134, 135). However, the target of IgM autoantibodies is unknown, and immunoblotting with recombinant protein of BP180 C-terminal domain showed multiple non-specific bands (136). IgM is mainly associated with BP caused by lupus erythematosus (132); however, it is rarely associated with BP due to infections (137), macroglobulinemia (136, 138), and surgical factors (139). The presence of IgM autoantibodies seems to not influence the course or outcome of the disease; and the role of IgM autoantibodies in the pathophysiology of BP remains elusive.

## The Cleavage and Depletion of BP180

Followed by various autoantibody-mediated inflammatory responses, the BP180 cleavage and depletion have been proposed as the terminal effect that causes reduced adhesion and blister formation. *In vitro*, the cleavage and shedding of BP180 ectodomain is an event related to detachment, migration, proliferation, differentiation, and wound healing of keratinocytes (50, 140–144). Generally, the cleaved ectodomain does not generate pathogenic epitopes. However, excessive cleavage, shedding, or depletion can lead to reduced adhesion and blister formation.

**Figure 5:**
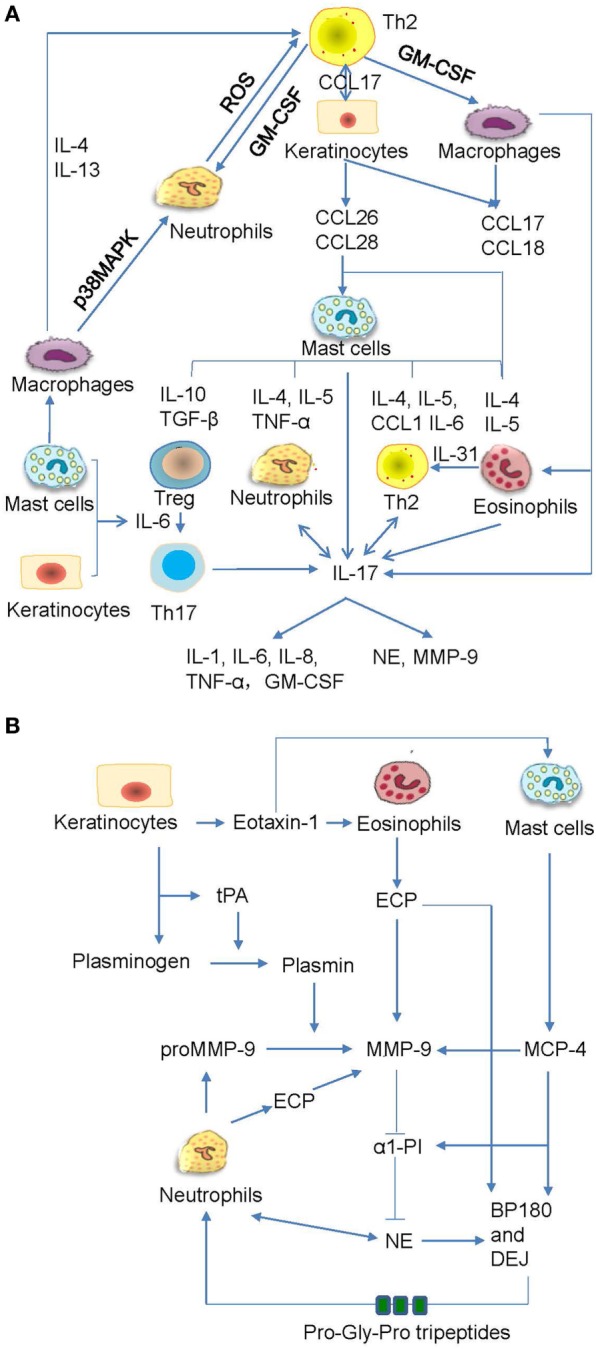
The crosstalk networks of the inflammatory cells and the proteases. **(A)** The autoactive Th2 cells secrete type II cytokines, which act on macrophages, keratinocytes, and fibroblasts and induce the chemotaxis of mast cells. The mast cells produce various cytokines and chemokines, which act on the regulatory T (Treg) cells, neutrophils, Th2, eosinophils, and macrophages. All these inflammatory cells can release interleukin (IL)-17, which not only act on neutrophils and Th2 but also prompt inflammatory response and proteases release. **(B)** The keratinocytes synthesize tissue plasmogen activator (tPA), which activates plasmin followed by the activation of matrix metalloproteinase (MMP)-9. The eosinophil cationic protein (ECP) and mast cell protease (MCP)-4 can also activate MMP-9. MMP-9 inhibits production of α1-proteinase inhibitor (α1-PI) while promote generation of neutrophil elastase (NE). All these proteases act on BP180 and dermal–epidermal junction (DEJ). The cleaved BP180 can release pro-gly-pro tripeptides and attract neutrophils.

Bullous pemphigoid autoantibody-induced infiltration of mast cells, eosinophils, and neutrophils can lead to the production of various inflammatory factors and proteases that contribute to the induction of blister formation. Increased levels of IL-1β, IL-2, IL-4, IL-5, IL-6, IL-8, IL-10, IL-13, IL-17, IL-22, IL-23, IL-31, IL-36, interferon-γ, tumor necrosis factor (TNF)-α, transforming growth factor-β, RANTES (regulated on activation, normal T cell expressed and secreted), monocyte chemotactic protein 1, interferon gamma-induced protein 10, and C-C chemokine ligand (CCL) 17 have been detected in skin lesions, serum, or blister fluid of BP patients (14, 19, 97, 145–150). In addition, C-C chemokine receptor 3 ligands, such as CCL 11, CCL13, CCL18, CCL26, and CCL28, have been shown to be increased in skin and/or sera of BP patients (43, 146, 151, 152). Increased levels of CCL1, CCL2, and chemokine C-X-C motif ligand-10 were detected in sera of BP patients (153, 154). Moreover, increasing data revealed their functional involvements in BP (97, 149, 151, 153, 155–158) (Figure 5A). The proteases produced by inflammatory cells are functionally involved as well (79, 159) (Figure 5B). The inflammatory cells can release mast cell protease (MCP)-4, matrix metalloproteinase (MMP)-9, neutrophil elastase (NE), plasmin, and eosinophil cationic protein (ECP), which cleave and degrade BP180, thus leading to dermal–epidermal separation and blister formation (20, 149, 157, 160–164) Pathogenic anti-BP180 IgG failed to induce subepidermal blistering in mice that were deficient in either NE or MMP-9 (89). MMP-9 can regulate NE activity by inactivating α1-proteinase inhibitor (α1-PI) (159). Furthermore, α1-PI serves as a chemoattractant for neutrophils once it is cleaved and exacerbates tissue damage (165). MMP-9 can also cleave BP180 into small tripeptides Pro-Gly-Pro, which significantly enhance neutrophil chemotaxis and NE release (149). These infiltrated cells also release IL-17, which significantly upregulates the production of MMP-9 and elastase in neutrophils (149, 166). The released IL-17 could, in turn, stimulate neutrophils to produce more IL-17 and form an amplified loop (167) (Figure 5A). Therefore, inflammatory factors and proteases induced by inflammatory cells play key roles in the cleavage and depletion of BP180, and targeting these inflammatory networks may be a promising therapeutic strategy in the treatment of BP.

However, BP180 cleavage may also occur in the absence of anti-BP180 autoantibodies (140). Such physiological cleavage is mediated by ADAMs (140). Our study further reveals that TNF-like weak inducer of apoptosis (TWEAK), which is a multifaceted cytokine that participates in various skin inflammatory responses, can exacerbate the BP180 reduction and keratinocyte adhesion (19). Moreover, the effect of TWEAK on BP180 cleavage involves the activation of extracellular signal-regulated kinase and nuclear factor-κB pathways as well as the downstream ADAMs, in which ADAM 8, 9, 10, 15, and 17 have been suggested to participate in BP180 cleavage or BP development (19, 168, 169). We also found high expression of MMP-9, ADAM9, ADAM10, and ADAM17 in BP lesions and in keratinocytes upon TWEAK/Fn14 activation (19). The upregulation of MMP-9 and ADAM10 is responsible for the shedding of membrane CD46, which further enhances BP180 NC16A IgG-mediated complement activation and blister formation (82). Therefore, the role of TWEAK in BP development can be mainly ascribed to the abnormally high expression of ADAMs and other proteases. By considering the absence or insignificant expression of TWEAK in noninvolved skin, we conclude that TWEAK likely plays a secondary inflammatory role rather than being a primary participant (19, 170). Further investigations are required to establish the clear-cut function of TWEAK in BP.

## Potential Therapeutic Targets

Considerable progress made by recent studies updated our understanding of BP pathogenesis. The availability of novel BP animal models provides important tools to further gain insights on the pathophysiology of the autoimmune disease. However, there is a limited progress regarding BP therapy. As BP180 is a molecule with multiple epitopes, a better insight on the mechanisms of immune responses induced by binding of autoantibodies to BP180 on different epitopes is crucial for the design of novel and more specific therapeutic strategies for this life-threatening autoimmune disorder (Table 1).

**Table 1:** Potential treatment targets for bullous pemphigoid (BP).

Categories	Targets	Drugs or methods	Potential effects	Reference
Immune tolerance	Regulatory T (Treg) cells	Interleukin (IL)-10	Increasing Treg cells	(171)
		Low-dose IL-2	Inducing significant Treg cells expansion	(172, 173)
		Oxymatrine	Upregulating FOXP3 Treg cells and reducing the production of tumor necrosis factor-α and IL-17A	(174)
	BP180 NC16A	Gene gun delivery of NC16a-encoding DNA	Inducting tolerance of BP180	(175)
	BP180	Lactic-co-glycolic acid nanoparticles	Inducing antigen-specific T cell tolerance	(176)

B cells	CD20	Rituximab	Reducing all subclasses of immunoglobulin G (IgG) anti-BP180 autoantibodies	(102, 177)
		Rituximab and intravenous immunoglobulin	Producing a prolonged and sustained remission in patients with active and recalcitrant BP	(39, 178)
		Calcineurin inhibitors	Suppressing naive B cells	(179)

T cells	CD25	Anti-CD25 antibodies	Targeting IL-2 receptor on activated T cells	(180)
	Calcineurin	Calcineurin inhibitors	Inhibiting nuclear factor of activated T cells and blocking T-cell-dependent production of IgG	(181)
	CD4+ T cells	IL-10	Lowering the number of circulating CD4+ T cells	(171)

Co-stimulators	BAFF–APRIL	Tabaluma (anti-BAFF)	Neutralizing autoreactive and memory B cells	(182)
		Anti-APRIL	Anti-proliferation and reducing autoantibodies production	(183, 184)
	CD40–CD40L	Anti-CD40	Regulating both innate and adaptive immunity and the activation of antigen-specific T cells	(185)

Autoantibodies	IgG	SM101	A soluble FcγR that competes with the interaction of IgG with membrane FcγRs	(186)
	IgE	Omalizumab	Inhibiting IgE binding to FcεRI	(108)
	Autoantibodies	Immunoadsorption	Declining the serum autoantibody levels	(187, 188)

*APRIL, a proliferation-inducing ligand; BAFF, B-cell activating factor*.

### The Recovery of Immune Tolerance

Targeting immune tolerance is a coveted approach for the treatment of various autoimmune diseases, as current treatment options often involve non-specific immunosuppression. BP is closely associated with the disturbance of self-tolerance, in which the reduction in Treg cells plays a key role. Therefore, the increase in Treg cells will help to recover immune tolerance and prevent BP development. Previously, recombinant IL-10 has been used to increase circulating Treg cells and to lower CD4+ T cells (171). The use of low-dose recombinant IL-2 could also induce significant expansion of Treg cells *in vivo* and preferentially restore Treg cells (172). Low-dose IL-2-induced Treg cell proliferation is subsequently followed by increased programmed cell death 1 (PD-1) expression (173). PD-1 inhibitor causes BP eruptions, thus suggesting the value of targeting PD-1 upregulation in BP treatment (102, 189). Oxymatrine, a monosomic alkaloid extracted from the Chinese herb *Sophora flavescens* Ait, can upregulate FOXP3+ Treg cells and reduce the production of TNF-α and IL-17A, thus aiding in the recovery of immune tolerance (174). Previously, nanotechnology is therapeutically used to inhibit the detrimental immune responses in autoimmunity through its direct immunosuppressive effect on antigen-presenting cells B and T cells, or indirectly by delivering compounds that result in immunotolerance (190). Gene gun delivery of NC16A-encoding DNA on gold particles results in Treg cell-mediated tolerance to BP180 (175). Antigen-coupled biodegradable poly (lactic-co-glycolic acid) nanoparticles have been used to induce antigen-specific T cell tolerance, which is a promising method that targets organ-specific BP (176). All aforementioned methods could improve immune tolerance and block the potential production of autoantibodies.

### Therapeutic Prevention of Excessive Antibody Production

Targeting the effector B and T cells to prevent the production of “pathogenic” autoantibodies may be a promising method in BP treatment. Rituximab used for depleting CD20+ B cells can reduce all subclasses of anti-BP180 IgG antibodies and has shown efficacy in case reports of patients with refractory BP (39, 177, 178). Autoreactive T cells are also associated with IgG autoantibodies production. Targeting autoreactive T cells using anti-CD25 antibodies and calcineurin inhibitors could modulate immune responses (181, 191). Anti-CD25 antibodies bind to high-affinity heterotrimeric IL-2 receptor on activated T cells, block the IL-2/IL-2 receptor signaling, and inhibit the propagation of T cell activation, thereby limiting the damaging effects of further T cell recruitment in autoimmune diseases (180). Calcineurin can dephosphorylate and inhibit nuclear factors of activated T cells and regulate T-cell activation and differentiation (181). The inhibition of nuclear factors of activated T cells may directly suppress skin injuries by blocking T-cell-dependent production of IgG, as IgG deposition is central to the development of bullae in BP. Additionally, the interaction between T and B cells needs co-stimulatory factors. Hence, targeting co-stimulatory molecules using special monoclonal antibodies could also disrupt the interaction of T and B cells and block the synthesis of autoantibodies (182–185, 192). For pathogen-induced BP, the suppression of dendritic cell-mediated autoimmunity or toll-like receptor antagonist is also practicable (193, 194).

### Neutralization of Pathogenic Antibodies

Immunoglobulin G autoantibodies are the main pathogenetic antibodies that act on FcγR to induce blister production. SM101, a soluble FcγR, competes with the interaction of IgG and membrane FcγRs and prevents the development of BP (186). Omalizumab, which targets IgE autoantibodies, can neutralize the activity of IgE in BP and control the disease activity (108). Furthermore, therapies targeting IgE–mast cells–eosinophils/basophils interaction may also demonstrate promising results in the treatment of BP (112). Moreover, immunoadsorption with high-affinity matrices that selectively bind to human IgG and IgE provides an alternative way of removing autoantibodies (187, 195).

### Prospective

Despite the complexity and diversity of the dermatosis, there is still hope for BP patients. Novel promising agents targeting different mechanisms of BP development are necessary. In addition, a multifactorial animal model for BP is warranted as well, and it should mimic not only the presence of specific pathogenic autoantibodies but also the additional triggers, such as environmental factors, medications, comorbid conditions, and infections, in disease initiation. Furthermore, future investigations are required as there may be the presence of unidentified antigenic epitopes that are indispensable for disease development.

## Conclusion

Bullous pemphigoid has been regarded as a well-characterized, organ-specific, mainly anti-BP180 autoantibody-mediated blistering skin disorder. Both IgG and IgE play vital roles in BP development *via* complement-dependent or -independent inflammatory pathways. However, the roles of IgA and IgM are still uncertain, and further investigation is needed. Knowledge of the BP180 target sites and of the interaction between BP180 and anti-BP180 autoantibodies is pivotal for the exploration of novel and more specific therapeutic methods so as to reduce BP morbidity and mortality. The translation of bench findings into bedside strategies for the treatment of this complex disease still remains to be a challenge. Although BP180-based therapy appears not to be close at hand yet, a better understanding of the role of BP180 would further approximate that to practice.

## Author Contributions

YL and YX conceived this paper. YL and LL wrote this manuscript. All the authors read and approved the final manuscript.

## Conflict of Interest Statement

The authors declare that the research was conducted in the absence of any commercial or financial relationships that could be construed as a potential conflict of interest.
